# Structural and Genetic Diversity of Lysis Modules in Bacteriophages Infecting the Genus *Streptococcus*

**DOI:** 10.3390/genes16070842

**Published:** 2025-07-19

**Authors:** Mathilde Saint-Jean, Olivier Claisse, Claire Le Marrec, Johan Samot

**Affiliations:** 1 CHU Bordeaux, Department of Dentistry and Oral Health, University of Bordeaux, F-33000 Bordeaux, France; mathilde.saint-jean@u-bordeaux.fr; 2 University of Bordeaux, INRAE, Bordeaux INP, Bordeaux Sciences Agro, UMR 1366, OENO, ISVV, F-33140 Villenave d’Ornon, France; olivier.claisse@u-bordeaux.fr (O.C.); clehenaff@enscbp.fr (C.L.M.); 3 CHU Bordeaux, Department of Oral Surgery, University of Bordeaux, INRAE, Bordeaux INP, Bordeaux Sciences Agro, UMR 1366, OENO, ISVV, F-33000 Bordeaux, France

**Keywords:** *Streptococcus*, bacteriophages, endolysins, holins, computational biology, microbiology

## Abstract

**Background/Objectives**: Bacteriophages infecting the genus *Streptococcus* play a crucial role in microbial ecology and have potential applications in biotechnology and medicine. Despite their importance, significant gaps remain in our understanding of their lysis modules. This study aims to address these deficiencies by analyzing the genomic diversity and lysis module organization in *Streptococcus* phages. **Methods**: A search was conducted in the NCBI RefSeq database to identify phage genomes infecting *Streptococcus*. A representative panel was selected based on taxonomic diversity. Lysis modules were annotated and visualized, functional domains in endolysins were identified, and holins were characterized. **Results**: A total of 205 phage genomes were retrieved from the NCBI RefSeq database, of which 185 complete genomes were analyzed. A subset of 34 phages was selected for in-depth analysis, ensuring the representation of taxonomic diversity. The lysis modules were annotated and visualized, revealing five distinct organizations. Among the 256 identified endolysins, 25 distinct architectural organizations were observed, with amidase activity being the most prevalent. Holins were classified into 9 of the 74 families listed in the Transporter Classification Database, exhibiting one to three transmembrane domains. **Conclusions**: This study provides insights into the structural diversity of lysis modules in *Streptococcus* phages, paving the way for future research and potential biotechnological applications.

## 1. Introduction

Antimicrobial resistance (AMR) poses a major global health threat, with antibiotic-resistant bacterial infections estimated to have caused 4.95 million deaths worldwide in 2019 [1]. In response to this growing crisis, phage therapy has re-emerged as a promising alternative or complement to traditional antibiotics.

Bacteriophages, or phages, are viruses that infect bacteria with high specificity, often targeting a single species or even a specific strain [2]. They can be broadly classified as lytic or temperate: lytic phages rapidly lyse and kill their bacterial hosts, whereas temperate phages may integrate into the bacterial genome as prophages. Numerous clinical cases have demonstrated the therapeutic potential of lytic phages, particularly against multi-drug-resistant bacterial infections [3].

One of the most promising components of phage therapy is the exploitation of the lysis module, a set of genes that orchestrate bacterial cell wall degradation at the end of the phage replication cycle. In phages infecting Gram-positive bacteria, such as *Streptococcus*, the lysis module typically comprises an endolysin, which enzymatically cleaves the peptidoglycan, and a holin, which forms pores in the cytoplasmic membrane to facilitate endolysin access. In contrast, phages infecting Gram-negative hosts often include an additional component, the spanin complex (Rz and Rz1 gene products), responsible for disrupting the outer membrane after peptidoglycan degradation [4]. Variants of this system also exist: some phages use pinholins, which create small lesions in the membrane to activate SAR (Signal–Arrest–Release) endolysins, a type of endolysin tethered to the membrane and released upon membrane depolarization [5]. These mechanistic variations underscore the functional diversity and evolutionary specialization of phage lysis strategies.

Endolysins, in particular, have drawn attention for their high specificity, being able to target selected bacterial genera, species, or even strains [6]. Their therapeutic potential has been notably demonstrated against pathogens such as *Streptococcus* spp. [7,8,9]. Bacteria from this genus are involved in a broad spectrum of diseases, ranging from mild infections to life-threatening conditions such as pneumonia and meningitis [10,11]. The increasing prevalence of antibiotic-resistant *Streptococcus* strains highlights the urgent need for alternative treatment options [12,13,14]. Notably, endolysins have also shown efficacy in eliminating intracellular *Streptococcus*, further expanding their clinical relevance [8].

Beyond their clinical importance, some *Streptococcus* spp., such as *Streptococcus thermophilus*, hold significant industrial value due to their use in fermented dairy products like yogurt and cheese, underlining their biotechnological and economic importance [15].

This study aims to conduct a comparative in silico analysis of the lysis modules encoded by phages infecting *Streptococcus* spp. By examining the genetic organization, domain architecture, and functional diversity of holins as well as endolysins, we seek to provide insights into their functional variability and potential for therapeutic application.

## 2. Materials and Methods

### 2.1. Retrieval of Phages Infecting the Streptococcus Genus

A search was performed in the NCBI RefSeq database [16] on 7 March 2025, using the keywords “*Streptococcus* phage” and “*Streptococcus* bacteriophage.” Only complete and annotated genomes were retained. Duplicates and evidently incomplete sequences were manually verified and excluded. When available, the bacterial host species was recorded.

### 2.2. Selection of a Representative Panel

Taxonomic classification (class, family, subfamily, genus, and species) was retrieved from the International Committee on Taxonomy of Viruses (ICTV) database [17] on 22 April 2025. For genera represented by multiple phages, a representative genome was selected based on citation frequency in the literature or the diversity/complexity of the lysis module. Phages unassigned at the genus level were systematically included. The phylogenetic diversity of the final panel was assessed using genome-based distance analyses with VICTOR [18] and VIRIDIC [19]. The resulting tree was visualized using iTOL v6 [20].

### 2.3. Detection and Annotation of Lysis Module

Phage genomes from the selected panel were annotated using Pharokka v1.7.5 [21] and visualized with LoVis4u version 0.1.2 [22] to identify genes associated with the lysis module. Annotations were cross-checked against RefSeq data. In ambiguous cases, unannotated proteins were analyzed using BLASTp [23] to determine potential homology to known holins or endolysins.

### 2.4. Detection of Functional Domains in Endolysins

Endolysin sequences were analyzed with phiBIOTICS [24] and SMART [25] to detect enzymatically active domains (EADs) and cell wall-binding domains (CBDs). Additional searches were conducted using HMMER, the Conserved Domain Database (CDD) [26], and MOTIF Search (https://www.genome.jp/tools/motif/, accessed on 28 May 2025), incorporating domain models from Pfam, COG, TIGRFAM, and SMART. An E-value cutoff of <0.01 was applied.

### 2.5. Characterization of Holins

Identified holins were submitted to BLASTp against the Transporter Classification Database [27] for functional classification. Transmembrane domain (TMD) number and topology were predicted using DeepTMHMM v1.0.42 [28] and confirmed via Protter version 1.0 [29].

## 3. Results

### 3.1. Diversity of Phages Infecting the Streptococcus Genus

**Figure 1:**
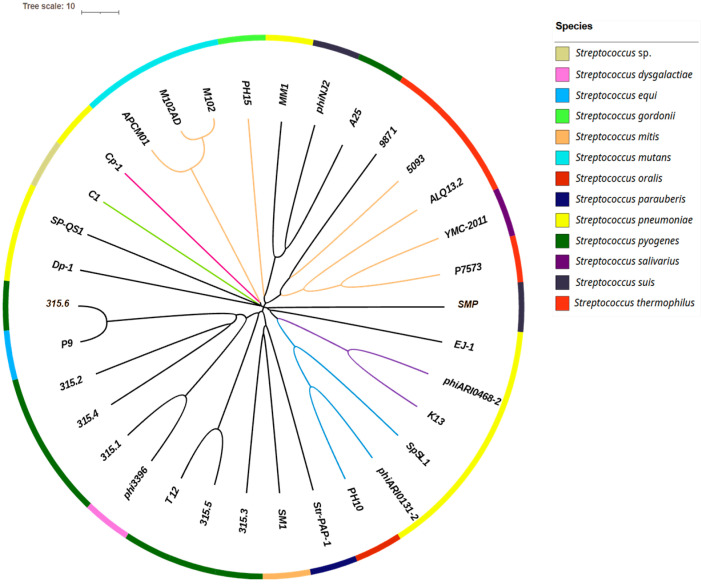
Phylogenetic tree of the 34 phages selected for their taxonomic diversity. The tree was constructed using pairwise nucleotide alignments between phage genomes, based on a distance matrix computed by VIRIDIC and visualized with iTOL. Branches are colored according to the phages’ taxonomic classification. At the family level: *Aliceevansviridae* (orange), *Madridviridae* (fuchsia), and *Rountreeviridae* (green); at the subfamily level: *Ferrettivirinae* (purple) and *Mcshanvirinae* (blue). Branches shown in black indicate phages for which neither the family nor the subfamily is known. Bacterial hosts are represented by colored squares (as indicated in the color key), forming an outer ring.

A total of 205 *Streptococcus*-associated phages were initially retrieved from RefSeq. After excluding incomplete entries, 185 complete genomes were retained; their NCBI accession numbers are listed in Appendix A.1. A subset of 34 phages was selected for in-depth analysis, representing the observed taxonomic diversity (Figure 1). This selection included phages belonging to different genera when such classification was available, or otherwise phages that remained unclassified at the genus level according to the ICTV database. These 34 phages collectively infect 12 distinct *Streptococcus* species, although the host species remained unidentified for 1 of them. Notably, 20 phages in this subset were unassigned at the genus level. The four major groups of *S. thermophilus*-infecting phages were included: Pac (*Brussowvirus*), Cos (*Moineauvirus*), 5093 (*Vansinderenvirus*), and 9871 (*Piorkowskivirus*) (Appendix A.2).

### 3.2. Organization of Lysis Modules

**Figure 2:**
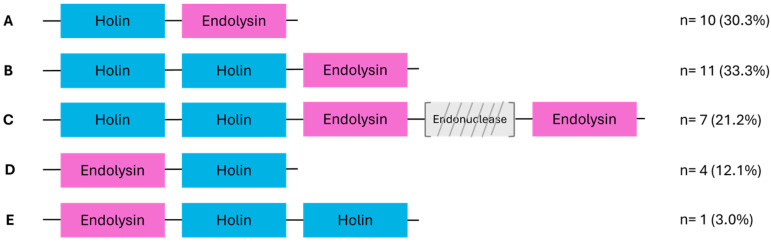
Schematic representation of the different lysis module organizations identified among the analyzed phages (*n* = 33). Among the 34 phages examined, 33 harbored a recognizable lysis module. Five distinct organizations, labeled A to E, were defined based on the number and arrangement of lysis-related genes, holins (blue) and endolysins (pink). The number of phages exhibiting each organization is indicated on the right. In organizations A to C, holin genes precede endolysins, whereas in D and E, endolysins come first. Organization C includes an endonuclease gene (gray, diagonally striped) that is variably present between holin and endolysin. Each module is represented in the same transcriptional direction (left to right).

Gene annotations revealed the presence of holin and endolysin genes, enabling the delineation of lysis modules in the 34 selected phages (Appendix A.3). Five distinct module organizations (A to E) were identified (Figure 2). These modules contain up to two ORFs each for holins and endolysins.

In 85% of phages, holin-encoding ORFs preceded endolysin-encoding ones, while this order was reversed in the remaining 15%. Lysis genes were generally contiguous, though some modules featured intercalated genes. For instance, phage ALQ13.2 (*Brussowvirus*) harbored an endonuclease and a hypothetical protein between two endolysins. Phage SP-QS1 uniquely lacked a clearly defined lysis module, with holin and endolysin genes separated by 18 unrelated genes, mostly encoding structural or hypothetical proteins.

### 3.3. Endolysin Architecture

**Figure 3:**
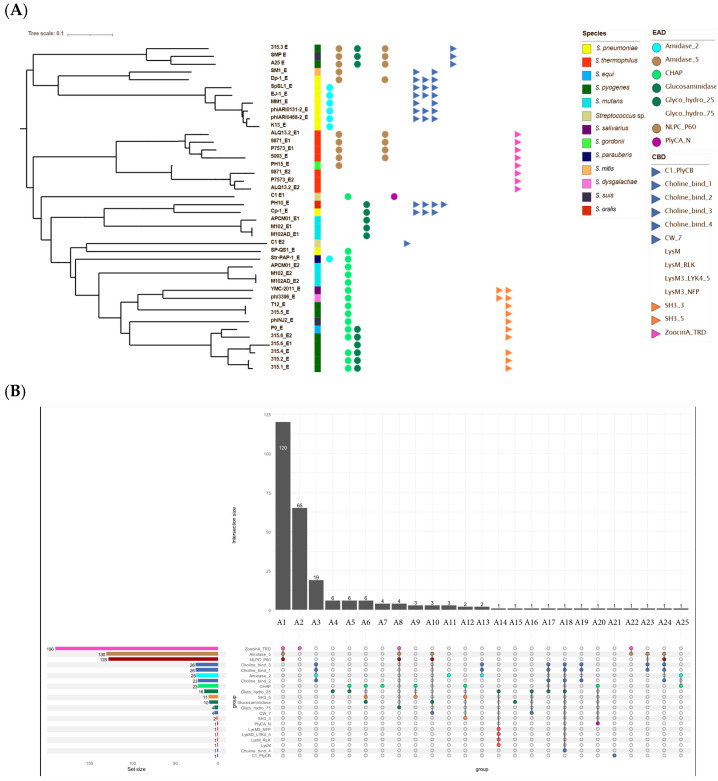
Diversity and domain architecture of phage endolysins. (**A**) Phylogenetic tree of the 42 endolysins identified among the 34 selected phages. The tree was built using amino acid sequences aligned with ClustalW pairwise alignment, a slow algorithm, and the BLOSUM matrix). Endolysins are named using the abbreviated name of the originating phage, with the suffix _E when a single endolysin is present, or _E1/_E2 in the case of phages encoding two. For each endolysin, EADs and CBDs, when identified, are represented by colored circles and triangles, respectively (domain color codes are indicated in the figure). Colored squares indicate the bacterial species targeted by the phages encoding these endolysins. (**B**) Upset plot of the 256 analyzed endolysins. The plot displays the 25 distinct combinations of enzymatic and binding domains, labeled A1 to A25, based on the presence and arrangement of known EADs and CBDs. Each vertical bar represents the number of endolysins sharing a specific combination, while the connected dots below each bar indicate the motifs involved in that combination. On the left, a horizontal bar chart shows the overall frequency of each individual domain motif across all endolysins. A color code is used to distinguish motifs, with similar motifs sharing the same color.

Among the 185 phages, 114 encoded a single endolysin, while 71 encoded two. Endolysin genes ranged from 228 to 1386 bp, yielding a total of 256 distinct endolysins. Within the 34-phage subset, 26 had a single endolysin (Figure 3A).

These 256 proteins exhibited 25 architectural types, defined by EAD and CBD composition and arrangement (Figure 3B, Appendix A.4). The most common catalytic activity was amidase (Amidase_5 domain), and the most frequent CBD was ZoocinA_TRD. Seven architectures (A4, A5, A7, A11, A15, A20, and A25) lacked identifiable CBDs; three (A2, A19, and A21) lacked catalytic domains. Architectures were numbered by decreasing frequency (A1 = most common). Some were unique (A14–A25), and five (A5, A8, A14, A16, and A19) were absent from the representative subset.

Architectures lacking catalytic domains were usually co-encoded with catalytically active endolysins. For example, A2 (ZoocinA_TRD only) consistently co-occurred with A1 (Amidase_5 & NLPC_P60 & ZoocinA_TRD). Similarly, A20 (Chap & PlyCA) and A21 (PlyCB) were both encoded by phage C1, reflecting the PlyC endolysin structure.

An exception was phage IPP55, where a lone endolysin belonged to architecture A19. Further analysis using CDD and BLASTP revealed two conserved domains: a PGRP superfamily motif and a pneumo_PspA domain. The protein shared > 99% identity with *S. pneumoniae* LytA amidase (WP_073176617.1).

Notably, all three *S. mutans* phages encoding two endolysins lacked any identifiable CBDs.

Generally, catalytic domains were located at the N-terminus, with CBDs at the C-terminus. An exception was architecture A10, where CW7 motifs interrupted the Amidase_5/NLPC_P60 and Glucosaminidase domains. In some endolysins combining Amidase_5 and NLPC_P60, overlapping genes were observed, with the latter starting ~39 nt downstream of the former.

### 3.4. Characterization of Holins

**Figure 4:**
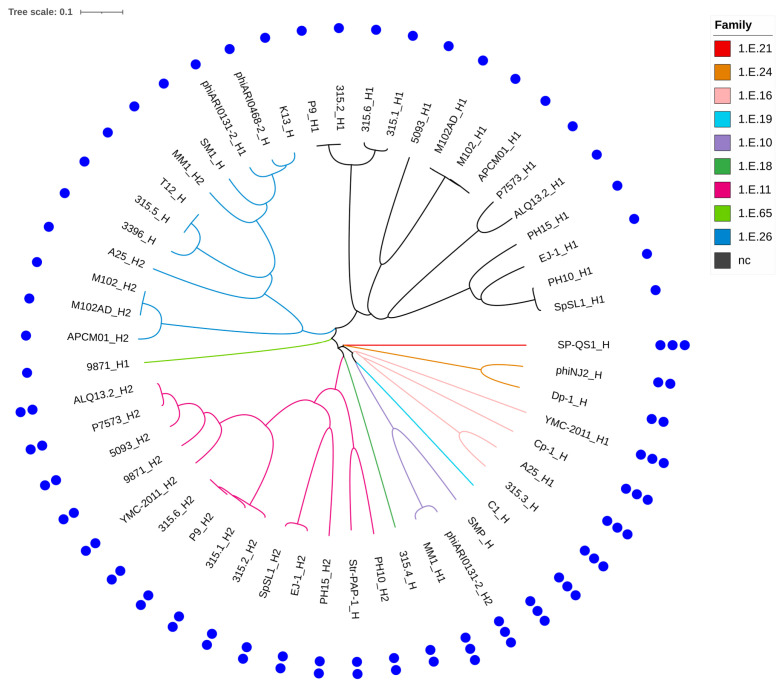
Phylogenetic tree of the 53 holins identified among the 34 selected phages. The tree was built using amino acid sequences aligned with ClustalW (pairwise alignment, slow algorithm, BLOSUM matrix). Each holin is named using the abbreviated phage name, followed by the suffix _H when a single holin is present, or _H1/_H2 when two are encoded. Nine holin families were identified and are color-coded on the tree branches. Black branches indicate holins with no assigned family. The number of predicted transmembrane domains is shown by blue dots forming the outer ring of the tree.

A total of 340 holins were identified among the 185 phages analyzed. Within the subset of 34 phages, 53 holins were detected, distributed across 9 families (1.E.10, 1.E.11, 1.E.16, 1.E.18, 1.E.19, 1.E.21, 1.E.24, 1.E.26, and 1.E.65) out of the 74 currently listed in the Transporter Classification Database (TCDB). Fourteen holins remained unclassified. Phages encoding two holins always featured proteins from different families, consistent with divergent evolutionary origins (Figure 4).

The number of predicted TMDs was also assessed for all 53 holins and ranged from one to three, depending on the family. The number of TMDs was conserved within each family. Holins from families 1.E.26 and 1.E.65, as well as the unclassified holins, each contained a single TMD. Holins from families 1.E.11, 1.E.18, and 1.E.24 harbored two TMDs, whereas those from the remaining four families exhibited three TMDs.

Interestingly, among the 19 phages encoding two holins, only those infecting *S. mutans* featured holins with a single TMD in both cases.

## 4. Discussion

This study highlights significant deficiencies in the current taxonomic annotation of *Streptococcus* phages, with many genomes, particularly within the selected subset, remaining unclassified at the genus level. The lysis modules exhibited considerable diversity, including instances of intercalated ORFs such as endonucleases. Endolysin genomic sequences were generally short, even though they sometimes encode two structurally distinct proteins. This diversity appears to be facilitated by gene overlapping. Amidase activity was the most prevalent enzymatic function observed. While most endolysin gene architectures conformed to the typical N-terminal EAD and C-terminal CBD layout, some deviated from this pattern, including cases with a central CBD or a separate CBD-encoding ORF. Some CBDs also remained uncharacterized. Holins were predominantly found in tandem across the studied phages, although this trend was less pronounced in the subset. The diversity of holins was relatively limited, with those in the subset classified into only 9 of the 74 families currently listed in TCDB, and exhibiting between one and three transmembrane domains depending on the family.

Although all 185 phages were assigned to the class *Caudoviricetes*, the taxonomic information for several phages, particularly those in the subset, remains incomplete. This is hardly surprising given the trends reported by Turner et al. [30]. Viral taxonomy evolves rapidly, and the ICTV acknowledges that taxonomic assignment may remain partial for certain viruses, particularly when based solely on genomic data without cultivation, or in the context of metagenomic studies [31]. Other factors also complicate viral taxonomy, such as deficiencies in the curation of deposited viral genomes, especially for prophages. For example, phage phiNIH 1.1 (NC_003157.5) shares near-complete genomic identity with phage 315.4 (NC_004587.1), and, according to VIRIDIC analysis (data not shown), should arguably be classified within the same species. The only distinguishing feature is the presence of a gene (NC_003157.5:149-1255) encoding a peptide-methionine (R)-S-oxide reductase (MsrB), located upstream of the integrase gene in phiNIH 1.1 and likely acquired from the *S. pyogenes* host genome. These challenges underscore the need for improved viral genome curation, and some have proposed the use of artificial intelligence to assist with this task [32].

The findings from this study on the lysis modules of phages infecting *Streptococcus* species led to the proposal of a classification based on the position and number of ORFs encoding lysins and holins. This classification could be extended to phages infecting Gram-negative bacteria by incorporating the number and position of ORFs encoding spanins [5]. Although lysis-related genes are often contiguous, interspersed ORFs are not uncommon. Among these, endonucleases have been reported, under the designation *lysin intergenic locus* [33].

Regarding endolysin genomic sequences, the analysis revealed a notable rate of gene overlapping, allowing a single ORF to encode two distinct enzymatic functions. The most common association involved amidase activity (Amidase_5 domain) and endopeptidase activity (NLPC_P60 domain). This genomic arrangement supports the hypothesis of viral genome compression via overprinting, a mechanism widely used by phages to optimize coding efficiency [34,35].

Amidase activity emerged as the dominant enzymatic function in the endolysins of phages infecting the genus *Streptococcus*, in contrast with endolysins from *Staphylococcus* phages, where Cysteine, Histidine-dependent Amidohydrolases/Peptidases (CHAP) domains often predominate and amidase domains may play an auxiliary role, possibly contributing to peptidoglycan binding rather than catalysis [36]. This distinction may reflect functional adaptations to differences in host peptidoglycan structure and susceptibility.

The organization of endolysins observed here aligns with the classical architecture described for phages infecting Gram-positive bacteria, typically featuring an N-terminal EAD and a C-terminal CBD [37,38]. However, previously reported exceptions were also identified in this study, such as the well-characterized endolysin PlyC, whose CBD (PlyCB) oligomerizes into an octamer [33], or the presence of a central CBD flanked by EADs at both termini, as described for phage LambdaSa2 lysin and PlySK1249 lysin [39,40]. In some endolysins, particularly from phages infecting *S. mutans*, only EADs could be identified, which is consistent with a previous review [41]. The 25 architectural organizations described here therefore represent only a subset of the 89 known architectures [42,43].

The frequent presence of tandem holins supports previous observations by Escobedo et al. [44], who reported that the presence of a second holin may improve lytic activity. Nevertheless, further studies are needed to clarify the functional significance of membrane domain organization in holin regulation and activity, as current classification systems do not fully reflect the mechanistic diversity of these proteins [45,46].

## 5. Conclusions

This study revealed extensive diversity in the lysis modules of *Streptococcus* phages, with gene overlap and interspersed ORFs contributing to their structural complexity. These findings pave the way for future investigations, including the functional characterization of unannotated CBDs and the refinement of lysin engineering strategies for therapeutic purposes.

## Figures and Tables

**Figure A1:**
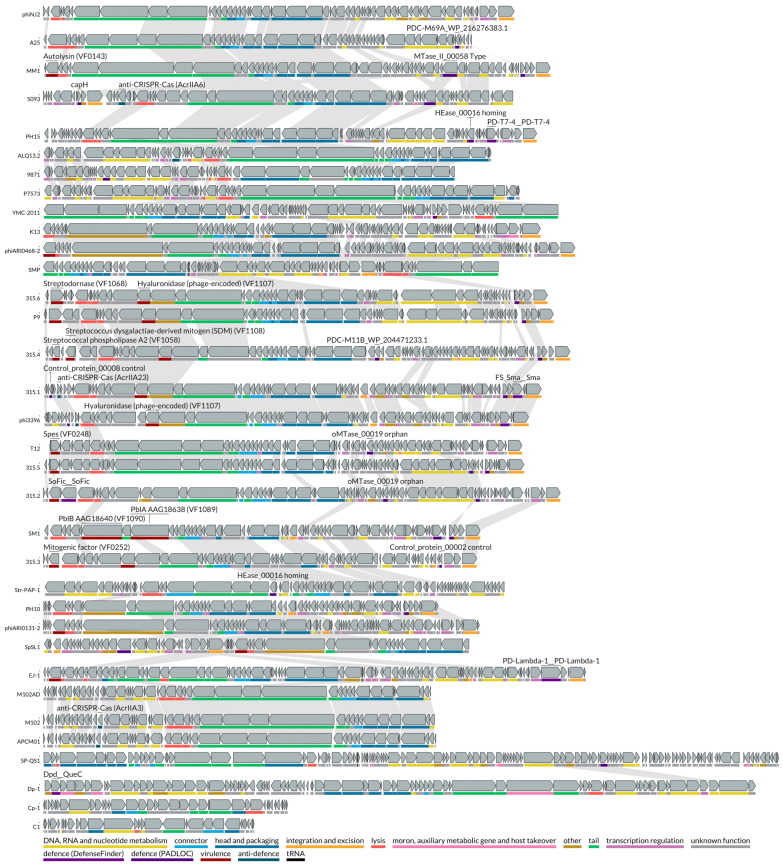
Nucleotide alignment of the 34 selected phages. Alignment was performed using the Lovis4u softwarev0.0.12. ORF functions are shown using a color-coded scheme.

## Data Availability

The original contributions presented in this study are included in the article. Further inquiries can be directed to the corresponding author.

## Abbreviations

The following abbreviations are used in this manuscript:

CBD	Cell-wall Binding Domain
CDD	Conserved Domain Database
CHAP	Cysteine, Histidine-dependent Amidohydrolases/Peptidases
EAD	Enzymatically Active Domain
ICTV	International Committee on Taxonomy of Viruses
ORF	Open Reading Frame
SAR	Signal–Arrest–Release
TCDB	Transporter Classification Database
TMD	Transmembrane domain

## Appendix A

### Appendix A.1

**Table A1:** List of the 185 phages included in the study.

Phage Name	Bacterial Target	NCBI RefSeq	kbp	Class	Family	Subfamily	Genus	Species
*Streptococcus* phage 20617	*Streptococcus thermophilus*	NC_023503.1	48	*Caudoviricetes*	*Aliceevansviridae*		*Brussowvirus*	*Brussowvirus 20617*
*Streptococcus* phage ALQ13.2	*Streptococcus thermophilus*	NC_013598.1	35	*Caudoviricetes*	*Aliceevansviridae*		*Brussowvirus*	*Brussowvirus ALQ132*
*Streptococcus* phage 2972	*Streptococcus thermophilus*	NC_007019.1	34	*Caudoviricetes*	*Aliceevansviridae*		*Brussowvirus*	*Brussowvirus bv2972*
*Streptococcus* phage 858	*Streptococcus thermophilus*	NC_010353.1	35	*Caudoviricetes*	*Aliceevansviridae*		*Brussowvirus*	*Brussowvirus bv858*
*Streptococcus* phage 01205	*Streptococcus thermophilus*	NC_004303.1	43	*Caudoviricetes*	*Aliceevansviridae*		*Brussowvirus*	*Brussowvirus bvO1205*
*Streptococcus* phage CHPC1042	*Streptococcus thermophilus*	NC_071061.1	42	*Caudoviricetes*	*Aliceevansviridae*		*Brussowvirus*	*Brussowvirus CHPC1042*
*Streptococcus* phage CHPC1109	*Streptococcus thermophilus*	NC_071062.1	33	*Caudoviricetes*	*Aliceevansviridae*		*Brussowvirus*	*Brussowvirus CHPC1109*
*Streptococcus* phage CHPC1152	*Streptococcus thermophilus*	NC_071063.1	35	*Caudoviricetes*	*Aliceevansviridae*		*Brussowvirus*	*Brussowvirus CHPC1152*
*Streptococcus* phage CHPC1230	*Streptococcus thermophilus*	NC_071064.1	39	*Caudoviricetes*	*Aliceevansviridae*		*Brussowvirus*	*Brussowvirus CHPC1230*
*Streptococcus* phage CHPC1246	*Streptococcus thermophilus*	NC_071065.1	36	*Caudoviricetes*	*Aliceevansviridae*		*Brussowvirus*	*Brussowvirus CHPC1246*
*Streptococcus* phage CHPC1247	*Streptococcus thermophilus*	NC_071066.1	35	*Caudoviricetes*	*Aliceevansviridae*		*Brussowvirus*	*Brussowvirus CHPC1247*
*Streptococcus* phage CHPC1248	*Streptococcus thermophilus*	NC_071067.1	38	*Caudoviricetes*	*Aliceevansviridae*		*Brussowvirus*	*Brussowvirus CHPC1248*
*Streptococcus* phage CHPC640	*Streptococcus thermophilus*	NC_071068.1	40	*Caudoviricetes*	*Aliceevansviridae*		*Brussowvirus*	*Brussowvirus CHPC640*
*Streptococcus* phage CHPC869	*Streptococcus thermophilus*	NC_071069.1	37	*Caudoviricetes*	*Aliceevansviridae*		*Brussowvirus*	*Brussowvirus CHPC869*
*Streptococcus* phage CHPC931	*Streptococcus thermophilus*	NC_071070.1	36	*Caudoviricetes*	*Aliceevansviridae*		*Brussowvirus*	*Brussowvirus CHPC931*
*Streptococcus* phage CHPC951	*Streptococcus thermophilus*	NC_071071.1	36	*Caudoviricetes*	*Aliceevansviridae*		*Brussowvirus*	*Brussowvirus CHPC951*
*Streptococcus* phage CHPC952	*Streptococcus thermophilus*	NC_071072.1	36	*Caudoviricetes*	*Aliceevansviridae*		*Brussowvirus*	*Brussowvirus CHPC952*
*Streptococcus* phage P4761	*Streptococcus thermophilus*	NC_071073.1	38	*Caudoviricetes*	*Aliceevansviridae*		*Brussowvirus*	*Brussowvirus P4761*
*Streptococcus* phage P7571	*Streptococcus thermophilus*	NC_071074.1	34	*Caudoviricetes*	*Aliceevansviridae*		*Brussowvirus*	*Brussowvirus P7571*
*Streptococcus* phage P7951	*Streptococcus thermophilus*	NC_071075.1	34	*Caudoviricetes*	*Aliceevansviridae*		*Brussowvirus*	*Brussowvirus P7951*
*Streptococcus* phage P7952	*Streptococcus thermophilus*	NC_071076.1	37	*Caudoviricetes*	*Aliceevansviridae*		*Brussowvirus*	*Brussowvirus P7952*
*Streptococcus* phage P7953	*Streptococcus thermophilus*	NC_071077.1	35	*Caudoviricetes*	*Aliceevansviridae*		*Brussowvirus*	*Brussowvirus P7953*
*Streptococcus* phage P7954	*Streptococcus thermophilus*	NC_071078.1	35	*Caudoviricetes*	*Aliceevansviridae*		*Brussowvirus*	*Brussowvirus P7954*
*Streptococcus* phage P7955	*Streptococcus thermophilus*	NC_071079.1	36	*Caudoviricetes*	*Aliceevansviridae*		*Brussowvirus*	*Brussowvirus P7955*
*Streptococcus* phage P9853	*Streptococcus thermophilus*	NC_071080.1	34	*Caudoviricetes*	*Aliceevansviridae*		*Brussowvirus*	*Brussowvirus P9853*
*Streptococcus* phage Sfi11	*Streptococcus thermophilus*	NC_002214.1	39	*Caudoviricetes*	*Aliceevansviridae*		*Brussowvirus*	*Brussowvirus Sfi11*
*Streptococcus* phage SW1151	*Streptococcus thermophilus*	NC_071081.1	35	*Caudoviricetes*	*Aliceevansviridae*		*Brussowvirus*	*Brussowvirus SW1151*
*Streptococcus* phage SW13	*Streptococcus thermophilus*	NC_071082.1	36	*Caudoviricetes*	*Aliceevansviridae*		*Brussowvirus*	*Brussowvirus SW13*
*Streptococcus* phage SW14	*Streptococcus thermophilus*	NC_071083.1	35	*Caudoviricetes*	*Aliceevansviridae*		*Brussowvirus*	*Brussowvirus SW14*
*Streptococcus* phage SW15	*Streptococcus thermophilus*	NC_071084.1	34	*Caudoviricetes*	*Aliceevansviridae*		*Brussowvirus*	*Brussowvirus SW15*
*Streptococcus* phage SW18	*Streptococcus thermophilus*	NC_071085.1	34	*Caudoviricetes*	*Aliceevansviridae*		*Brussowvirus*	*Brussowvirus SW18*
*Streptococcus* phage TP-778L	*Streptococcus thermophilus*	NC_022776.1	41	*Caudoviricetes*	*Aliceevansviridae*		*Brussowvirus*	*Brussowvirus TP778L*
*Streptococcus* phage TP-J34	*Streptococcus thermophilus*	NC_020197.1	45	*Caudoviricetes*	*Aliceevansviridae*		*Brussowvirus*	*Brussowvirus TPJ34*
MAG: *Streptococcus* phage VS-2018a	*Streptococcus thermophilus*	NC_071086.1	39	*Caudoviricetes*	*Aliceevansviridae*		*Brussowvirus*	*Brussowvirus VS2018a*
*Streptococcus* phage Abc2	*Streptococcus thermophilus*	NC_013645.1	34	*Caudoviricetes*	*Aliceevansviridae*		*Moineauvirus*	*Moineauvirus Abc2*
*Streptococcus* phage B0	*Streptococcus thermophilus*	NC_070655.1	36	*Caudoviricetes*	*Aliceevansviridae*		*Moineauvirus*	*Moineauvirus B0*
*Streptococcus* phage CHPC1005	*Streptococcus thermophilus*	NC_070656.1	37	*Caudoviricetes*	*Aliceevansviridae*		*Moineauvirus*	*Moineauvirus CHPC1005*
*Streptococcus* phage CHPC1027	*Streptococcus thermophilus*	NC_070657.1	35	*Caudoviricetes*	*Aliceevansviridae*		*Moineauvirus*	*Moineauvirus CHPC1027*
*Streptococcus* phage CHPC1029	*Streptococcus thermophilus*	NC_070658.1	35	*Caudoviricetes*	*Aliceevansviridae*		*Moineauvirus*	*Moineauvirus CHPC1029*
*Streptococcus* phage CHPC1033	*Streptococcus thermophilus*	NC_070659.1	36	*Caudoviricetes*	*Aliceevansviridae*		*Moineauvirus*	*Moineauvirus CHPC1033*
*Streptococcus* phage CHPC1036	*Streptococcus thermophilus*	NC_070660.1	36	*Caudoviricetes*	*Aliceevansviridae*		*Moineauvirus*	*Moineauvirus CHPC1036*
*Streptococcus* phage CHPC1041	*Streptococcus thermophilus*	NC_070661.1	38	*Caudoviricetes*	*Aliceevansviridae*		*Moineauvirus*	*Moineauvirus CHPC1041*
*Streptococcus* phage CHPC1045	*Streptococcus thermophilus*	NC_070662.1	34	*Caudoviricetes*	*Aliceevansviridae*		*Moineauvirus*	*Moineauvirus CHPC1045*
*Streptococcus* phage CHPC1046	*Streptococcus thermophilus*	NC_070663.1	34	*Caudoviricetes*	*Aliceevansviridae*		*Moineauvirus*	*Moineauvirus CHPC1046*
*Streptococcus* phage CHPC1062	*Streptococcus thermophilus*	NC_070664.1	40	*Caudoviricetes*	*Aliceevansviridae*		*Moineauvirus*	*Moineauvirus CHPC1062*
*Streptococcus* phage CHPC1067	*Streptococcus thermophilus*	NC_070665.1	34	*Caudoviricetes*	*Aliceevansviridae*		*Moineauvirus*	*Moineauvirus CHPC1067*
*Streptococcus* phage CHPC1091	*Streptococcus thermophilus*	NC_070666.1	37	*Caudoviricetes*	*Aliceevansviridae*		*Moineauvirus*	*Moineauvirus CHPC1091*
*Streptococcus* phage CHPC1148	*Streptococcus thermophilus*	NC_070667.1	39	*Caudoviricetes*	*Aliceevansviridae*		*Moineauvirus*	*Moineauvirus CHPC1148*
*Streptococcus* phage CHPC1156	*Streptococcus thermophilus*	NC_070668.1	34	*Caudoviricetes*	*Aliceevansviridae*		*Moineauvirus*	*Moineauvirus CHPC1156*
*Streptococcus* phage CHPC572	*Streptococcus thermophilus*	NC_070669.1	37	*Caudoviricetes*	*Aliceevansviridae*		*Moineauvirus*	*Moineauvirus CHPC572*
*Streptococcus* phage CHPC595	*Streptococcus thermophilus*	NC_070670.1	35	*Caudoviricetes*	*Aliceevansviridae*		*Moineauvirus*	*Moineauvirus CHPC595*
*Streptococcus* phage CHPC642	*Streptococcus thermophilus*	NC_070671.1	35	*Caudoviricetes*	*Aliceevansviridae*		*Moineauvirus*	*Moineauvirus CHPC642*
*Streptococcus* phage CHPC663	*Streptococcus thermophilus*	NC_070672.1	38	*Caudoviricetes*	*Aliceevansviridae*		*Moineauvirus*	*Moineauvirus CHPC663*
*Streptococcus* phage CHPC873	*Streptococcus thermophilus*	NC_070673.1	38	*Caudoviricetes*	*Aliceevansviridae*		*Moineauvirus*	*Moineauvirus CHPC873*
*Streptococcus* phage CHPC875	*Streptococcus thermophilus*	NC_070674.1	36	*Caudoviricetes*	*Aliceevansviridae*		*Moineauvirus*	*Moineauvirus CHPC875*
*Streptococcus* phage CHPC877	*Streptococcus thermophilus*	NC_070675.1	39	*Caudoviricetes*	*Aliceevansviridae*		*Moineauvirus*	*Moineauvirus CHPC877*
*Streptococcus* phage CHPC879	*Streptococcus thermophilus*	NC_070676.1	36	*Caudoviricetes*	*Aliceevansviridae*		*Moineauvirus*	*Moineauvirus CHPC879*
*Streptococcus* phage CHPC919	*Streptococcus thermophilus*	NC_070677.1	37	*Caudoviricetes*	*Aliceevansviridae*		*Moineauvirus*	*Moineauvirus CHPC919*
*Streptococcus* phage CHPC925	*Streptococcus thermophilus*	NC_070678.1	34	*Caudoviricetes*	*Aliceevansviridae*		*Moineauvirus*	*Moineauvirus CHPC925*
*Streptococcus* phage CHPC927	*Streptococcus thermophilus*	NC_070679.1	37	*Caudoviricetes*	*Aliceevansviridae*		*Moineauvirus*	*Moineauvirus CHPC927*
*Streptococcus* phage CHPC928	*Streptococcus thermophilus*	NC_070680.1	34	*Caudoviricetes*	*Aliceevansviridae*		*Moineauvirus*	*Moineauvirus CHPC928*
*Streptococcus* phage CHPC930	*Streptococcus thermophilus*	NC_070681.1	36	*Caudoviricetes*	*Aliceevansviridae*		*Moineauvirus*	*Moineauvirus CHPC930*
*Streptococcus* phage CHPC950	*Streptococcus thermophilus*	NC_070682.1	35	*Caudoviricetes*	*Aliceevansviridae*		*Moineauvirus*	*Moineauvirus CHPC950*
*Streptococcus* phage CHPC979	*Streptococcus thermophilus*	NC_070683.1	34	*Caudoviricetes*	*Aliceevansviridae*		*Moineauvirus*	*Moineauvirus CHPC979*
*Streptococcus* phage D1024	*Streptococcus thermophilus*	NC_070684.1	37	*Caudoviricetes*	*Aliceevansviridae*		*Moineauvirus*	*Moineauvirus D1024*
*Streptococcus* phage D1811	*Streptococcus thermophilus*	NC_070685.1	34	*Caudoviricetes*	*Aliceevansviridae*		*Moineauvirus*	*Moineauvirus D1811*
*Streptococcus* phage D4276	*Streptococcus thermophilus*	NC_070686.1	39	*Caudoviricetes*	*Aliceevansviridae*		*Moineauvirus*	*Moineauvirus D4276*
*Streptococcus* phage DT1	*Streptococcus thermophilus*	NC_002072.2	34	*Caudoviricetes*	*Aliceevansviridae*		*Moineauvirus*	*Moineauvirus DT1*
*Streptococcus* phage L5A1	*Streptococcus thermophilus*	NC_070687.1	36	*Caudoviricetes*	*Aliceevansviridae*		*Moineauvirus*	*Moineauvirus L5A1*
*Streptococcus* phage M19	*Streptococcus thermophilus*	NC_070688.1	34	*Caudoviricetes*	*Aliceevansviridae*		*Moineauvirus*	*Moineauvirus M19*
*Streptococcus* phage MM25	*Streptococcus thermophilus*	NC_070689.1	34	*Caudoviricetes*	*Aliceevansviridae*		*Moineauvirus*	*Moineauvirus MM25*
*Streptococcus* phage 128	*Streptococcus thermophilus*	NC_070651.1	34	*Caudoviricetes*	*Aliceevansviridae*		*Moineauvirus*	*Moineauvirus mv128*
*Streptococcus* phage 53	*Streptococcus thermophilus*	NC_070652.1	34	*Caudoviricetes*	*Aliceevansviridae*		*Moineauvirus*	*Moineauvirus mv53*
*Streptococcus* phage 7201	*Streptococcus thermophilus*	NC_002185.1	35	*Caudoviricetes*	*Aliceevansviridae*		*Moineauvirus*	*Moineauvirus mv7201*
*Streptococcus* phage 73	*Streptococcus thermophilus*	NC_070653.1	36	*Caudoviricetes*	*Aliceevansviridae*		*Moineauvirus*	*Moineauvirus mv73*
*Streptococcus* phage 7A5	*Streptococcus thermophilus*	NC_070654.1	35	*Caudoviricetes*	*Aliceevansviridae*		*Moineauvirus*	*Moineauvirus mv7A5*
*Streptococcus* phage P0091	*Streptococcus thermophilus*	NC_070690.1	35	*Caudoviricetes*	*Aliceevansviridae*		*Moineauvirus*	*Moineauvirus P0091*
*Streptococcus* phage P3681	*Streptococcus thermophilus*	NC_070691.1	36	*Caudoviricetes*	*Aliceevansviridae*		*Moineauvirus*	*Moineauvirus P3681*
*Streptococcus* phage P3684	*Streptococcus thermophilus*	NC_070692.1	35	*Caudoviricetes*	*Aliceevansviridae*		*Moineauvirus*	*Moineauvirus P3684*
*Streptococcus* phage P5641	*Streptococcus thermophilus*	NC_070693.1	37	*Caudoviricetes*	*Aliceevansviridae*		*Moineauvirus*	*Moineauvirus P5641*
*Streptococcus* phage P5651	*Streptococcus thermophilus*	NC_070694.1	34	*Caudoviricetes*	*Aliceevansviridae*		*Moineauvirus*	*Moineauvirus P5651*
*Streptococcus* phage P7132	*Streptococcus thermophilus*	NC_070695.1	35	*Caudoviricetes*	*Aliceevansviridae*		*Moineauvirus*	*Moineauvirus P7132*
*Streptococcus* phage P7133	*Streptococcus thermophilus*	NC_070696.1	33	*Caudoviricetes*	*Aliceevansviridae*		*Moineauvirus*	*Moineauvirus P7133*
*Streptococcus* phage P7134	*Streptococcus thermophilus*	NC_070697.1	34	*Caudoviricetes*	*Aliceevansviridae*		*Moineauvirus*	*Moineauvirus P7134*
*Streptococcus* phage P7151	*Streptococcus thermophilus*	NC_070698.1	35	*Caudoviricetes*	*Aliceevansviridae*		*Moineauvirus*	*Moineauvirus P7151*
*Streptococcus* phage P7152	*Streptococcus thermophilus*	NC_070699.1	35	*Caudoviricetes*	*Aliceevansviridae*		*Moineauvirus*	*Moineauvirus P7152*
*Streptococcus* phage P7154	*Streptococcus thermophilus*	NC_070700.1	34	*Caudoviricetes*	*Aliceevansviridae*		*Moineauvirus*	*Moineauvirus P7154*
*Streptococcus* phage P7573	*Streptococcus thermophilus*	NC_070701.1	37	*Caudoviricetes*	*Aliceevansviridae*		*Moineauvirus*	*Moineauvirus P7573*
*Streptococcus* phage P7574	*Streptococcus thermophilus*	NC_070702.1	36	*Caudoviricetes*	*Aliceevansviridae*		*Moineauvirus*	*Moineauvirus P7574*
*Streptococcus* phage P7601	*Streptococcus thermophilus*	NC_070703.1	35	*Caudoviricetes*	*Aliceevansviridae*		*Moineauvirus*	*Moineauvirus P7601*
*Streptococcus* phage P7602	*Streptococcus thermophilus*	NC_070704.1	37	*Caudoviricetes*	*Aliceevansviridae*		*Moineauvirus*	*Moineauvirus P7602*
*Streptococcus* phage P7631	*Streptococcus thermophilus*	NC_070705.1	36	*Caudoviricetes*	*Aliceevansviridae*		*Moineauvirus*	*Moineauvirus P7631*
*Streptococcus* phage P7632	*Streptococcus thermophilus*	NC_070706.1	35	*Caudoviricetes*	*Aliceevansviridae*		*Moineauvirus*	*Moineauvirus P7632*
*Streptococcus* phage P7633	*Streptococcus thermophilus*	NC_070707.1	35	*Caudoviricetes*	*Aliceevansviridae*		*Moineauvirus*	*Moineauvirus P7633*
*Streptococcus* phage P9851	*Streptococcus thermophilus*	NC_070708.1	35	*Caudoviricetes*	*Aliceevansviridae*		*Moineauvirus*	*Moineauvirus P9851*
*Streptococcus* phage P9854	*Streptococcus thermophilus*	NC_070709.1	36	*Caudoviricetes*	*Aliceevansviridae*		*Moineauvirus*	*Moineauvirus P9854*
*Streptococcus* phage P9901	*Streptococcus thermophilus*	NC_070710.1	35	*Caudoviricetes*	*Aliceevansviridae*		*Moineauvirus*	*Moineauvirus P9901*
*Streptococcus* phage P9902	*Streptococcus thermophilus*	NC_070711.1	35	*Caudoviricetes*	*Aliceevansviridae*		*Moineauvirus*	*Moineauvirus P9902*
*Streptococcus* phage P9903	*Streptococcus thermophilus*	NC_070712.1	35	*Caudoviricetes*	*Aliceevansviridae*		*Moineauvirus*	*Moineauvirus P9903*
*Streptococcus* phage Sfi19	*Streptococcus thermophilus*	NC_000871.1	37	*Caudoviricetes*	*Aliceevansviridae*		*Moineauvirus*	*Moineauvirus Sfi19*
*Streptococcus* phage Sfi21	*Streptococcus thermophilus*	NC_000872.1	40	*Caudoviricetes*	*Aliceevansviridae*		*Moineauvirus*	*Moineauvirus Sfi21*
*Streptococcus* phage STP1	*Streptococcus thermophilus*	NC_070713.1	35	*Caudoviricetes*	*Aliceevansviridae*		*Moineauvirus*	*Moineauvirus STP1*
*Streptococcus* phage SW1	*Streptococcus thermophilus*	NC_070714.1	34	*Caudoviricetes*	*Aliceevansviridae*		*Moineauvirus*	*Moineauvirus SW1*
*Streptococcus* phage SW11	*Streptococcus thermophilus*	NC_070715.1	36	*Caudoviricetes*	*Aliceevansviridae*		*Moineauvirus*	*Moineauvirus SW11*
*Streptococcus* phage SW12	*Streptococcus thermophilus*	NC_070716.1	35	*Caudoviricetes*	*Aliceevansviridae*		*Moineauvirus*	*Moineauvirus SW12*
*Streptococcus* phage SW2	*Streptococcus thermophilus*	NC_070717.1	37	*Caudoviricetes*	*Aliceevansviridae*		*Moineauvirus*	*Moineauvirus SW2*
*Streptococcus* phage SW3	*Streptococcus thermophilus*	NC_070718.1	37	*Caudoviricetes*	*Aliceevansviridae*		*Moineauvirus*	*Moineauvirus SW3*
*Streptococcus* phage SW5	*Streptococcus thermophilus*	NC_070719.1	36	*Caudoviricetes*	*Aliceevansviridae*		*Moineauvirus*	*Moineauvirus SW5*
*Streptococcus* phage SW6	*Streptococcus thermophilus*	NC_070720.1	34	*Caudoviricetes*	*Aliceevansviridae*		*Moineauvirus*	*Moineauvirus SW6*
*Streptococcus* phage SW7	*Streptococcus thermophilus*	NC_070721.1	35	*Caudoviricetes*	*Aliceevansviridae*		*Moineauvirus*	*Moineauvirus SW7*
*Streptococcus* phage SW8	*Streptococcus thermophilus*	NC_070722.1	33	*Caudoviricetes*	*Aliceevansviridae*		*Moineauvirus*	*Moineauvirus SW8*
*Streptococcus* phage SWK3	*Streptococcus thermophilus*	NC_070723.1	35	*Caudoviricetes*	*Aliceevansviridae*		*Moineauvirus*	*Moineauvirus SWK3*
*Streptococcus* phage SWK6	*Streptococcus thermophilus*	NC_070724.1	34	*Caudoviricetes*	*Aliceevansviridae*		*Moineauvirus*	*Moineauvirus SWK6*
*Streptococcus* phage vB_SthS_VA214	*Streptococcus thermophilus*	NC_070725.1	38	*Caudoviricetes*	*Aliceevansviridae*		*Moineauvirus*	*Moineauvirus VA214*
*Streptococcus* phage vB_SthS_VA460	*Streptococcus thermophilus*	NC_070726.1	41	*Caudoviricetes*	*Aliceevansviridae*		*Moineauvirus*	*Moineauvirus VA460*
*Streptococcus* phage 5093	*Streptococcus thermophilus*	NC_012753.1	37	*Caudoviricetes*	*Aliceevansviridae*		*Vansinderenvirus*	*Vansinderenvirus 5093*
*Streptococcus* phage CHPC1198	*Streptococcus thermophilus*	NC_071053.1	33	*Caudoviricetes*	*Aliceevansviridae*		*Vansinderenvirus*	*Vansinderenvirus CHPC1198*
*Streptococcus* phage CHPC1151	*Streptococcus thermophilus*	NC_071057.1	33	*Caudoviricetes*	*Aliceevansviridae*		*Vansinderenvirus*	*Vansinderenvirus CHPC1232*
*Streptococcus* phage CHPC1282	*Streptococcus thermophilus*	NC_071058.1	34	*Caudoviricetes*	*Aliceevansviridae*		*Vansinderenvirus*	*Vansinderenvirus CHPC1282*
*Streptococcus* phage P0092	*Streptococcus thermophilus*	NC_071056.1	34	*Caudoviricetes*	*Aliceevansviridae*		*Vansinderenvirus*	*Vansinderenvirus P0092*
*Streptococcus* phage P0093	*Streptococcus thermophilus*	NC_071054.1	34	*Caudoviricetes*	*Aliceevansviridae*		*Vansinderenvirus*	*Vansinderenvirus P0093*
*Streptococcus* phage P0095	*Streptococcus thermophilus*	NC_071059.1	35	*Caudoviricetes*	*Aliceevansviridae*		*Vansinderenvirus*	*Vansinderenvirus P0095*
*Streptococcus* phage SW19	*Streptococcus thermophilus*	NC_071060.1	35	*Caudoviricetes*	*Aliceevansviridae*		*Vansinderenvirus*	*Vansinderenvirus SW19*
*Streptococcus* phage SW24	*Streptococcus thermophilus*	NC_071055.1	32	*Caudoviricetes*	*Aliceevansviridae*		*Vansinderenvirus*	*Vansinderenvirus SW24*
*Streptococcus* phage SW27	*Streptococcus thermophilus*	NC_071052.1	33	*Caudoviricetes*	*Aliceevansviridae*		*Vansinderenvirus*	*Vansinderenvirus SW27*
*Streptococcus* phage SW4	*Streptococcus thermophilus*	NC_071051.1	34	*Caudoviricetes*	*Aliceevansviridae*		*Vansinderenvirus*	*Vansinderenvirus SW4*
*Streptococcus* phage Cp-1	*Streptococcus pneumoniae*	NC_001825.1	19	*Caudoviricetes*	*Madridviridae*		*Cepunavirus*	*Cepunavirus Cp1*
*Streptococcus* phage Cp-7	*Streptococcus pneumoniae*	NC_042114.1	19	*Caudoviricetes*	*Madridviridae*		*Cepunavirus*	*Cepunavirus Cp7*
*Streptococcus* phage C1	Group C Streptococci	NC_004814.1	16	*Caudoviricetes*	*Rountreeviridae*		*Fischettivirus*	*Fischettivirus C1*
*Streptococcus* phage phiARI0004	*Streptococcus pneumoniae*	NC_031920.1	41	*Caudoviricetes*		*Ferrettivirinae*	*Hinxtonvirus*	*Hinxtonvirus ARI0004*
*Streptococcus* phage phiARI0031	*Streptococcus pneumoniae*	NC_031910.1	41	*Caudoviricetes*		*Ferrettivirinae*	*Hinxtonvirus*	*Hinxtonvirus ARI0031*
*Streptococcus* phage phiARI0462	*Streptococcus pneumoniae*	NC_031942.1	40	*Caudoviricetes*		*Ferrettivirinae*	*Hinxtonvirus*	*Hinxtonvirus ARI0062*
*Streptococcus* phage phiARI0468-1	*Streptococcus pneumoniae*	NC_031929.1	41	*Caudoviricetes*		*Ferrettivirinae*	*Hinxtonvirus*	*Hinxtonvirus ARI04681*
*Streptococcus* phage DCC1738	*Streptococcus pneumoniae*	NC_024361.1	38	*Caudoviricetes*		*Ferrettivirinae*	*Hinxtonvirus*	*Hinxtonvirus DCC1738*
*Streptococcus* phage IC1	*Streptococcus pneumoniae*	NC_024370.1	39	*Caudoviricetes*		*Ferrettivirinae*	*Hinxtonvirus*	*Hinxtonvirus IC1*
*Streptococcus* phage K13	*Streptococcus pneumoniae*	NC_024357.1	39	*Caudoviricetes*		*Ferrettivirinae*	*Hinxtonvirus*	*Hinxtonvirus K13*
*Streptococcus* phage phiARI0131-1	*Streptococcus pneumoniae*	NC_031901.1	42	*Caudoviricetes*		*Ferrettivirinae*	*Spinunavirus*	*Spinunavirus ARI01311*
*Streptococcus* phage phiARI0460-1	*Streptococcus pneumoniae*	NC_031913.1	41	*Caudoviricetes*		*Ferrettivirinae*	*Spinunavirus*	*Spinunavirus ARI04601*
*Streptococcus* phage phiARI0468-2	*Streptococcus pneumoniae*	NC_031923.1	42	*Caudoviricetes*		*Ferrettivirinae*	*Spinunavirus*	*Spinunavirus ARI04682*
*Streptococcus* phage phiARI0923	*Streptococcus pneumoniae*	NC_030946.1	33	*Caudoviricetes*		*Mcshanvirinae*	*Adrianbuildvirus*	*Adrianbuildvirus ARI0923*
*Streptococcus* phage SpSL1	*Streptococcus pneumoniae*	NC_027396.1	33	*Caudoviricetes*		*Mcshanvirinae*	*Adrianbuildvirus*	*Adrianbuildvirus SpSL1*
*Streptococcus* phage phiARI0131-2	*Streptococcus pneumoniae*	NC_031941.1	34	*Caudoviricetes*		*Mcshanvirinae*	*Medawarvirus*	*Medawarvirus ARI01312*
*Streptococcus* phage PH10	*Streptococcus oralis*	NC_012756.1	31	*Caudoviricetes*		*Mcshanvirinae*	*Phadecavirus*	*Phadecavirus PH10*
*Streptococcus* phage phiBHN167	*Streptococcus pneumoniae*	NC_022791.1	37	*Caudoviricetes*			*Paclarkvirus*	*Paclarkvirus BHN167*
*Streptococcus* phage IPP14	*Streptococcus pneumoniae*	NC_070925.1	37	*Caudoviricetes*			*Paclarkvirus*	*Paclarkvirus IPP14*
*Streptococcus* phage IPP39	*Streptococcus pneumoniae*	NC_070926.1	37	*Caudoviricetes*			*Paclarkvirus*	*Paclarkvirus IPP39*
*Streptococcus* phage IPP48	*Streptococcus pneumoniae*	NC_070928.1	37	*Caudoviricetes*			*Paclarkvirus*	*Paclarkvirus IPP48*
*Streptococcus* phage IPP52	*Streptococcus pneumoniae*	NC_070929.1	36	*Caudoviricetes*			*Paclarkvirus*	*Paclarkvirus IPP52*
*Streptococcus* phage IPP54	*Streptococcus pneumoniae*	NC_070923.1	38	*Caudoviricetes*			*Paclarkvirus*	*Paclarkvirus IPP54*
*Streptococcus* phage IPP55	*Streptococcus pneumoniae*	NC_070924.1	37	*Caudoviricetes*			*Paclarkvirus*	*Paclarkvirus IPP55*
*Streptococcus* phage IPP65	*Streptococcus pneumoniae*	NC_070922.1	39	*Caudoviricetes*			*Paclarkvirus*	*Paclarkvirus IPP65*
*Streptococcus* phage IPP66	*Streptococcus pneumoniae*	NC_070930.1	37	*Caudoviricetes*			*Paclarkvirus*	*Paclarkvirus IPP66*
*Streptococcus pneumoniae* bacteriophage MM1	*Streptococcus pneumoniae*	NC_003050.2	40	*Caudoviricetes*			*Paclarkvirus*	*Paclarkvirus MM1*
*Streptococcus* phage SpGS-1	*Streptococcus pneumoniae*	NC_070927.1	37	*Caudoviricetes*			*Paclarkvirus*	*Paclarkvirus SpGS-1*
*Streptococcus* phage CHPC577	*Streptococcus thermophilus*	NC_070901.1	35	*Caudoviricetes*			*Piorkowskivirus*	*Piorkowskivirus CHPC577*
*Streptococcus* phage CHPC926	*Streptococcus thermophilus*	NC_070900.1	30	*Caudoviricetes*			*Piorkowskivirus*	*Piorkowskivirus CHPC926*
*Streptococcus* virus 9871	*Streptococcus thermophilus*	NC_031069.1	32	*Caudoviricetes*			*Piorkowskivirus*	*Piorkowskivirus pv9871*
*Streptococcus* virus 9872	*Streptococcus thermophilus*	NC_031094.1	33	*Caudoviricetes*			*Piorkowskivirus*	*Piorkowskivirus pv9872*
*Streptococcus* virus 9874	*Streptococcus thermophilus*	NC_031023.1	32	*Caudoviricetes*			*Piorkowskivirus*	*Piorkowskivirus pv9874*
*Streptococcus* phage SW16	*Streptococcus thermophilus*	NC_070899.1	32	*Caudoviricetes*			*Piorkowskivirus*	*Piorkowskivirus SW16*
*Streptococcus* phage SW22	*Streptococcus thermophilus*	NC_070898.1	31	*Caudoviricetes*			*Piorkowskivirus*	*Piorkowskivirus SW22*
*Streptococcus* phage SP-QS1	*Streptococcus pneumoniae*	NC_021868.1	58	*Caudoviricetes*			*Saphexavirus*	*Saphexavirus SPQS1*
*Streptococcus* phage A25	*Streptococcus pyogenes*	NC_028697.1	33	*Caudoviricetes*			*Stonewallvirus*	*Stonewallvirus A25*
*Streptococcus* phage APCM01	*Streptococcus mutans*	NC_029030.1	31	*Caudoviricetes*				
*Streptococcus* phage Dp-1	*Streptococcus pneumoniae*	NC_015274.1	56	*Caudoviricetes*				
*Streptococcus* phage M102	*Streptococcus mutans*	NC_012884.1	31	*Caudoviricetes*				
*Streptococcus* phage M102AD	*Streptococcus mutans*	NC_028984.1	30	*Caudoviricetes*				
*Streptococcus* phage P9	*Streptococcus equi*	NC_009819.1	40	*Caudoviricetes*				
*Streptococcus* phage PH15	*Streptococcus gordonii*	NC_010945.1	39	*Caudoviricetes*				
*Streptococcus* phage phi3396	*Streptococcus dysgalactiae*	NC_009018.1	38	*Caudoviricetes*				
*Streptococcus* phage phiNIH1.1	*Streptococcus pyogenes*	NC_003157.5	43	*Caudoviricetes*				
*Streptococcus* phage phiNJ2	*Streptococcus suis*	NC_019418.1	37	*Caudoviricetes*				
*Streptococcus* phage SM1	*Streptococcus mitis*	NC_004996.1	34	*Caudoviricetes*				
*Streptococcus* phage SMP	*Streptococcus suis*	NC_008721.2	36	*Caudoviricetes*				
*Streptococcus* phage Str-PAP-1	*Streptococcus parauberis*	NC_028666.1	36	*Caudoviricetes*				
*Streptococcus* phage T12	*Streptococcus pyogenes*	NC_028700.1	37	*Caudoviricetes*				
*Streptococcus* phage YMC-2011	*Streptococcus salivarius*	NC_018285.1	40	*Caudoviricetes*				
*Streptococcus pyogenes* phage 315.3	*Streptococcus pyogenes*	NC_004586.1	34	*Caudoviricetes*				
*Streptococcus* virus 9873	*Streptococcus thermophilus*	NC_047763.1	32	*Caudoviricetes*				
*Streptococcus* prophage 315.1	*Streptococcus pyogenes*	NC_004584.1	39	*Caudoviricetes*				
*Streptococcus* prophage 315.2	*Streptococcus pyogenes*	NC_004585.1	41	*Caudoviricetes*				
*Streptococcus* prophage 315.4	*Streptococcus pyogenes*	NC_004587.1	41	*Caudoviricetes*				
*Streptococcus* prophage 315.5	*Streptococcus pyogenes*	NC_004588.1	38	*Caudoviricetes*				
*Streptococcus* prophage 315.6	*Streptococcus pyogenes*	NC_004589.1	40	*Caudoviricetes*				
*Streptococcus* prophage EJ-1	*Streptococcus pneumoniae*	NC_005294.1	42	*Caudoviricetes*				

Taxonomic information according to ICTV is provided when available.

### Appendix A.2

**Table A2:** List of the 34 selected phages.

Phage	Short Name for Figure Readability	Bacterial Target	NCBI RefSeq Accession	kbp	Class	Family	Subfamily	Genus	Species	
*Streptococcus* phage ALQ13.2	ALQ13.2	*Streptococcus thermophilus*	NC_013598.1	35	*Caudoviricetes*	*Aliceevansviridae*		*Brussowvirus*	*Brussowvirus ALQ132*	Pac group
*Streptococcus* phage P7573	P7573	*Streptococcus thermophilus*	NC_070701.1	37	*Caudoviricetes*	*Aliceevansviridae*		*Moineauvirus*	*Moineauvirus P7573*	Cos group
*Streptococcus* phage 5093	5093	*Streptococcus thermophilus*	NC_012753.1	37	*Caudoviricetes*	*Aliceevansviridae*		*Vansinderenvirus*	*Vansinderenvirus 5093*	5093 group
*Streptococcus* phage Cp-1	Cp-1	*Streptococcus pneumoniae*	NC_001825.1	19	*Caudoviricetes*	*Madridviridae*		*Cepunavirus*	*Cepunavirus Cp1*	
*Streptococcus* phage C1	C1	Group C Streptococci	NC_004814.1	16	*Caudoviricetes*	*Rountreeviridae*		*Fischettivirus*	*Fischettivirus C1*	
*Streptococcus* phage K13	K13	*Streptococcus pneumoniae*	NC_024357.1	39	*Caudoviricetes*		*Ferrettivirinae*	*Hinxtonvirus*	*Hinxtonvirus K13*	
*Streptococcus* phage phiARI0468-2	phiARI0468-2	*Streptococcus pneumoniae*	NC_031923.1	42	*Caudoviricetes*		*Ferrettivirinae*	*Spinunavirus*	*Spinunavirus ARI04682*	
*Streptococcus* phage SpSL1	SpSL1	*Streptococcus pneumoniae*	NC_027396.1	33	*Caudoviricetes*		*Mcshanvirinae*	*Adrianbuildvirus*	*Adrianbuildvirus SpSL1*	
*Streptococcus* phage phiARI0131-2	phiARI0131-2	*Streptococcus pneumoniae*	NC_031941.1	34	*Caudoviricetes*		*Mcshanvirinae*	*Medawarvirus*	*Medawarvirus ARI01312*	
*Streptococcus* phage PH10	PH10	*Streptococcus oralis*	NC_012756.1	31	*Caudoviricetes*		*Mcshanvirinae*	*Phadecavirus*	*Phadecavirus PH10*	
*Streptococcus pneumoniae* bacteriophage MM1	MM1	*Streptococcus pneumoniae*	NC_003050.2	40	*Caudoviricetes*			*Paclarkvirus*	*Paclarkvirus MM1*	
*Streptococcus* virus 9871	9871	*Streptococcus thermophilus*	NC_031069.1	32	*Caudoviricetes*			*Piorkowskivirus*	*Piorkowskivirus pv9871*	9871 group
*Streptococcus* phage SP-QS1	SP-QS1	*Streptococcus pneumoniae*	NC_021868.1	58	*Caudoviricetes*			*Saphexavirus*	*Saphexavirus SPQS1*	
*Streptococcus* phage A25	A25	*Streptococcus pyogenes*	NC_028697.1	33	*Caudoviricetes*			*Stonewallvirus*	*Stonewallvirus A25*	
*Streptococcus* phage APCM01	APCM01	*Streptococcus mutans*	NC_029030.1	31	*Caudoviricetes*	*Aliceevansviridae*	No ICTV assignation	No ICTV assignation	No ICTV assignation	
*Streptococcus* phage M102	M102	*Streptococcus mutans*	NC_012884.1	31	*Caudoviricetes*	*Aliceevansviridae*	No ICTV assignation	No ICTV assignation	No ICTV assignation	
*Streptococcus* phage M102AD	M102AD	*Streptococcus mutans*	NC_028984.1	30	*Caudoviricetes*	*Aliceevansviridae*	No ICTV assignation	No ICTV assignation	No ICTV assignation	
*Streptococcus* phage PH15	PH15	*Streptococcus gordonii*	NC_010945.1	39	*Caudoviricetes*	*Aliceevansviridae*	No ICTV assignation	No ICTV assignation	No ICTV assignation	
*Streptococcus* phage YMC-2011	YMC-2011	*Streptococcus salivarius*	NC_018285.1	40	*Caudoviricetes*	*Aliceevansviridae*	No ICTV assignation	No ICTV assignation	No ICTV assignation	
*Streptococcus* phage Dp-1	Dp-1	*Streptococcus pneumoniae*	NC_015274.1	56	*Caudoviricetes*	*No ICTV assignation*	No ICTV assignation	No ICTV assignation	No ICTV assignation	
*Streptococcus* prophage EJ-1	EJ-1	*Streptococcus pneumoniae*	NC_005294.1	42	*Caudoviricetes*	*No ICTV assignation*	No ICTV assignation	No ICTV assignation	No ICTV assignation	
*Streptococcus* phage P9	P9	*Streptococcus equi*	NC_009819.1	40	*Caudoviricetes*	*No ICTV assignation*	No ICTV assignation	No ICTV assignation	No ICTV assignation	
*Streptococcus* phage phi3396	phi3396	*Streptococcus dysgalactiae*	NC_009018.1	38	*Caudoviricetes*	*No ICTV assignation*	No ICTV assignation	No ICTV assignation	No ICTV assignation	
*Streptococcus* phage phiNJ2	phiNJ2	*Streptococcus suis*	NC_019418.1	37	*Caudoviricetes*	*No ICTV assignation*	No ICTV assignation	No ICTV assignation	No ICTV assignation	
*Streptococcus* phage SM1	SM1	*Streptococcus mitis*	NC_004996.1	34	*Caudoviricetes*	*No ICTV assignation*	No ICTV assignation	No ICTV assignation	No ICTV assignation	
*Streptococcus* phage SMP	SMP	*Streptococcus suis*	NC_008721.2	36	*Caudoviricetes*	*No ICTV assignation*	No ICTV assignation	No ICTV assignation	No ICTV assignation	
*Streptococcus* phage Str-PAP-1	Str-PAP-1	*Streptococcus parauberis*	NC_028666.1	36	*Caudoviricetes*	*No ICTV assignation*	No ICTV assignation	No ICTV assignation	No ICTV assignation	
*Streptococcus* phage T12	T12	*Streptococcus pyogenes*	NC_028700.1	37	*Caudoviricetes*	*No ICTV assignation*	No ICTV assignation	No ICTV assignation	No ICTV assignation	
*Streptococcus pyogenes* phage 315.3	315.3	*Streptococcus pyogenes*	NC_004586.1	34	*Caudoviricetes*	*No ICTV assignation*	No ICTV assignation	No ICTV assignation	No ICTV assignation	
*Streptococcus* prophage 315.1	315.1	*Streptococcus pyogenes*	NC_004584.1	39	*Caudoviricetes*	*No ICTV assignation*	No ICTV assignation	No ICTV assignation	No ICTV assignation	
*Streptococcus* prophage 315.2	315.2	*Streptococcus pyogenes*	NC_004585.1	41	*Caudoviricetes*	*No ICTV assignation*	No ICTV assignation	No ICTV assignation	No ICTV assignation	
*Streptococcus* prophage 315.4	315.4	*Streptococcus pyogenes*	NC_004587.1	41	*Caudoviricetes*	*No ICTV assignation*	No ICTV assignation	No ICTV assignation	No ICTV assignation	
*Streptococcus* prophage 315.5	315.5	*Streptococcus pyogenes*	NC_004588.1	38	*Caudoviricetes*	*No ICTV assignation*	No ICTV assignation	No ICTV assignation	No ICTV assignation	
*Streptococcus* prophage 315.6	315.6	*Streptococcus pyogenes*	NC_004589.1	40	*Caudoviricetes*	*No ICTV assignation*	No ICTV assignation	No ICTV assignation	No ICTV assignation	

The abbreviated names used in graphical representations are indicated.

### Appendix A.4

**Table A3:** Summary of the architectures identified among the 256 endolysins.

Architecture	Count	EAD	CBD	Phages List
A1	120	Amidase_5 & NLPC_P60	ZoocinA_TRD	CHPC1151_E1, CHPC572_E1, CHPC1027_E1, SW27_E1, P7134_E1, P0093_E1, P0092_E1, CHPC1282_E1, SW14_E1, CHPC1033_E1, SW12_E1, SW24_E, STP1_E1, SW6_E1, P7955_E1, **P7573_E1**, P7571_E1, P0091_E, L5A1_E, SW4_E1, P9854_E1, CHPC927_E1, CHPC926_E1, CHPC925_E1, SW1151_E1, CHPC950_E1, CHPC1248_E1, CHPC1247_E1, CHPC1246_E1, CHPC1230_E1, CHPC1046_E1, SW11_E1, CHPC877_E, 128_E, P7133_E, MM25_E, **5093_E**, CHPC979_E, CHPC928_E, P7602_E, P7633_E, P7601_E, P7632_E, P7631_E, SW7_E, P3684_E, CHPC875_E, CHPC1091_E, CHPC930_E, CHPC1067_E, 7A5_E, SW3_E, B0_E, P3681_E, SW15_E, P7574_E, SW19_E1, CHPC952_E, CHPC931_E1, CHPC869_E1, SW8_E1, P5651_E1, CHPC1029_E1, CHPC1042_E1, P9851_E1, P9853_E1, CHPC640_E1, P7152_E1, SW18_E1, 9874_E1, CHPC1041_E1, D4276_E1, P0095_E1, CHPC1005_E1, CHPC879_E, vB_SthS_VA460_E2, MAG-VS-2018a_E, CHPC663_E1, 20617_E, 858_E1, 2972_E1, P7151_E1, CHPC1156_E1, vB_SthS_VA214_E2, CHPC642_E, P7132_E, SWK3_E, CHPC1036_E, CHPC1045_E, SWK6_E, P7154_E, CHPC1148_E, 7201_E, CHPC1198_E1, CHPC577_E1, **9871_E1**, SW22_E1, SW16_E, 9873_E1, M19_E, 53_E, CHPC1152_E, SW5_E, P9901_E, SW2_E, CHPC951_E, Abc2_E, 73_E, D1811_E1, D1024_E1, **ALQ13.2_E1**, 9872_E1, DT1_E1, TP-778L_E, TP-J34_E, Sfi19_E, CHPC919_E, Sfi21_E, Sfi11_E, 1205_E
A2	65		ZoocinA_TRD	vB_SthS_VA460_E1, 858_E2, CHPC1246_E2, CHPC1230_E2, 9874_E2, CHPC663_E2, P7571_E2, CHPC1033_E2, CHPC1027_E2, CHPC931_E2, CHPC869_E2, SW4_E2, CHPC1282_E2, CHPC1198_E2, SW27_E2, SW11_E2, P9854_E2, P9851_E2, P7134_E2, P0093_E2, P0092_E2, CHPC1248_E2, CHPC1247_E2, CHPC1151_E2, 2972_E2, P9853_E2, CHPC926_E2, **9871_E2**, SW22_E2, SW1151_E2, CHPC1042_E2, CHPC1029_E2, vB_SthS_VA214_E1, P4761_E2, 9873_E2, 9872_E2, SW19_E2, D1811_E2, D1024_E2, CHPC595_E2, DT1_E2, CHPC1156_E2, CHPC927_E2, P0095_E2, CHPC925_E2, CHPC640_E2, CHPC1041_E2, SW14_E2, D4276_E2, SW8_E2, CHPC950_E2, CHPC1046_E2, SW12_E2, **ALQ13.2_E2**, P7151_E2, CHPC577_E2, STP1_E2, CHPC572_E2, P7955_E2, **P7573_E2**, SW6_E2, SW18_E2, P7152_E2, P5651_E2, CHPC1005_E2
A3	19	Amidase_2	Choline_bind_1 & Choline_bind_2 & Choline_bind_3	**SpSL1_E**, **EJ-1_E**, phiARI0462_E, phiARI0460-1_E, phiARI0131-1_E, **phiARI0131-2_E**, phiARI0923_E, phiARI0004_E, **phiARI0468-2_E**, phiARI0468-1_E, phiARI0031_E, IPP48_E, phiBHN167_E, IPP14_E, IPP52_E, IC1_E, IPP39_E, IPP54_E, SpGS-1_E
A4	6	Glyco_hydro_25		**APCM01_E1**, **M102_E1**, **M102AD_E1**, P7951_E, P5641_E, CHPC1062_E
A5	6	CHAP & Glyco_hydro_25		P7954_E, SW13_E, P7953_E, P7952_E, SW1_E, CHPC873_E
A6	6	Glucosaminidase & CHAP	SH3_5	**P9_E**, **315.6_E2**, **315.2_E**, **315.1_E**, phiNIH1.1_E, **315.4_E**
A7	4	CHAP		**SP-QS1_E**, **APCM01_E2**, **M102_E2**, **M102AD_E2**
A8	4	Amidase_5 & NLPC_P60 & Glyco_hydro_75	ZoocinA_TRD	P4761_E1, CHPC595_E1, P9903_E, P9902_E
A9	3	CHAP	SH3_5	**T12_E**, **315.5_E**, **phiNJ2_E**
A10	3	Amidase_5 & NLPC_P60 & Glucosaminidase	CW7	**315.3_E**, **SMP_E**, **A25_E**
A11	3	Amidase_2		**K13_E**, DCC1738_E, IPP66_E
A12	2	CHAP	SH3_3 & SH3_5	**YMC-2011_E**, **phi3396_E**
A13	2	Amidase_2	Choline_bind_1 & Choline_bind_3	**MM1_E**, IPP65_E
A14	1	Glyco_hydro_25	LysM & LysM_RLK & LysM3_LYK4_5 & LysM3_NFP	CHPC1109_E
A15	1	Glucosaminidase		**315.6_E1**
A16	1	Glyco_hydro_25	CW7	Cp-7_E
A17	1	Glyco_hydro_25	Choline_bind_1 & Choline_bind_2 & Choline_bind_3	**Cp-1_E**
A18	1	Glyco_hydro_25	Choline_bind_1 & Choline_bind_2 & Choline_bind_3 & Choline_bind_4	**PH10_E**
A19	1		Choline_bind_1 & Choline_bind_2 & Choline_bind_3	IPP55_E
A20	1	PlyCA_N & CHAP		**C1 E1**
A21	1		C1_PlyCB	**C1 E2**
A22	1	Amidase_5	ZoocinA_TRD	**PH15_E**
A23	1	Amidase_5	Choline_bind_1 & Choline_bind_3	**SM1_E**
A24	1	Amidase_5 & NLPC_P60	Choline_bind_1 & Choline_bind_2 & Choline_bind_3	**Dp-1_E**
A25	1	CHAP & Amidase_2		**Str-PAP-1_E**

Each identified endolysin is associated with its domain architecture. Endolysins from phages included in the 34-phage subset are highlighted in bold.
